# 2-Mesitylacetic acid

**DOI:** 10.1107/S1600536809051514

**Published:** 2009-12-04

**Authors:** Jiang-Sheng Li, Qi-Xi He, Peng-Yu Li

**Affiliations:** a School of Chemistry and Biological Engineering, Changsha University of Science & Technology, Changsha 410004, People’s Republic of China; b College of Chemistry and Chemical Engineering, Hunan University, Changsha 410082, People’s Republic of China

## Abstract

In the title compound, C_11_H_14_O_2_, the dihedral angle between the CCOO carboxyl unit and the benzene ring is 85.37 (7)°. In the crystal, the mol­ecules are linked into inversion dimers by pairs of O—H⋯O hydrogen bonds.

## Related literature

For background to carboxylic acids as supra­molecular synthons, see: Thalladi *et al.* (1996 ▶).
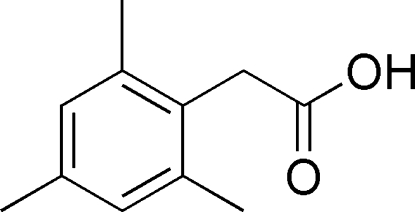


## Experimental

### 

#### Crystal data

C_11_H_14_O_2_
*M* *_r_* = 178.22
Monoclinic, 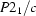

*a* = 8.2312 (16) Å
*b* = 15.366 (3) Å
*c* = 7.5708 (15) Å
β = 92.74 (3)°
*V* = 956.4 (3) Å^3^
*Z* = 4
Mo *K*α radiation
μ = 0.08 mm^−1^
*T* = 113 K
0.32 × 0.18 × 0.12 mm

#### Data collection

Rigaku Saturn CCD diffractometer
Absorption correction: multi-scan (*CrystalClear*; Rigaku/MSC, 2005 ▶) *T*_min_ = 0.974, *T*_max_ = 0.990
6163 measured reflections
1681 independent reflections
1259 reflections with *I* > 2σ(*I*)
*R*_int_ = 0.040

#### Refinement

*R*[*F* ^2^ > 2σ(*F* ^2^)] = 0.035
*wR*(*F* ^2^) = 0.104
*S* = 1.03
1681 reflections
123 parameters
H-atom parameters constrained
Δρ_max_ = 0.20 e Å^−3^
Δρ_min_ = −0.18 e Å^−3^

### 

Data collection: *CrystalClear* (Rigaku/MSC, 2005 ▶); cell refinement: *CrystalClear*; data reduction: *CrystalClear*; program(s) used to solve structure: *SHELXS97* (Sheldrick, 2008 ▶); program(s) used to refine structure: *SHELXL97* (Sheldrick, 2008 ▶); molecular graphics: *SHELXTL* (Sheldrick, 2008 ▶); software used to prepare material for publication: *SHELXL97*.

## Supplementary Material

Crystal structure: contains datablocks I, global. DOI: 10.1107/S1600536809051514/hb5261sup1.cif

Structure factors: contains datablocks I. DOI: 10.1107/S1600536809051514/hb5261Isup2.hkl

Additional supplementary materials:  crystallographic information; 3D view; checkCIF report

## Figures and Tables

**Table 1:** Hydrogen-bond geometry (Å, °)

*D*—H⋯*A*	*D*—H	H⋯*A*	*D*⋯*A*	*D*—H⋯*A*
O2—H2⋯O1^i^	0.82	1.84	2.6564 (15)	177

Symmetry code: (i) 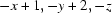.

**Fig. 1.**
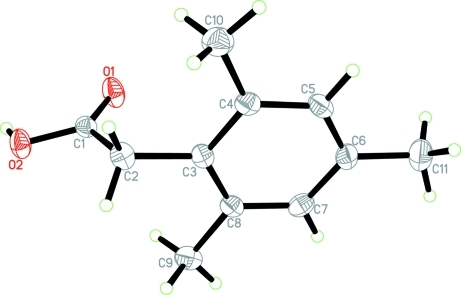
The molecule of (I) showing displacement ellipsoids drawn at the 50% probability level.

**Fig. 2.**
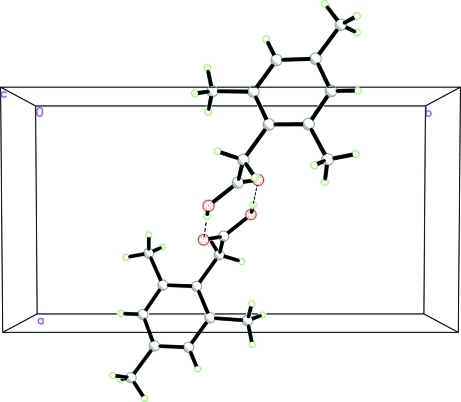
The dimer formed via intermolecular O—H···O hydrogen bonding.

## supplementary crystallographic information

### Comment

Carboxylic acid is a supramolecular synthon, widely used to construct supramolecular array with one to three different dimensions *via* hydrogen bonds (Thalladi *et al.*,  1996). Herein the structure of the title compound (I) is reported.

In the title molecule, (Fig 1), the carbonyl moiety C1/C2/O1/O2 forms an angle of 85.3797) with the benzene ring. In the crystal packing, molecules are linked into dimers by strong O—H···O H-bonding (Table 1 & Fig 2).

### Experimental

The title compound was available from Hunan institute of Chemical Industry, received without further purification.  Colourless blocks of (I) were obtained by evaporation from its solution of ethyl acetate/petroleum ether 1/4 (*v*/*v*).

### Refinement

All H atoms were positioned geometrically and constrained to ride on their parent atoms [C—H distances are 0.93 and 0.97Å with *U*_iso_(H) = 1.2 *U*_eq_(C) for aromatic and CH_2_ H atoms, 0.82 and 0.96Å with *U*_iso_ = 1.5*U*_eq_ (O and C) for OH and CH_3_ H atoms].

### Crystal data

**Table:**

C_11_H_14_O_2_	*F*(000) = 384
*M**_r_* = 178.22	*D*_x_ = 1.238 Mg m^−^^3^
Monoclinic, *P*2_1_/*c*	Melting point: 440-442 K K
Hall symbol: -P 2ybc	Mo *K*α radiation, λ = 0.71073 Å
*a* = 8.2312 (16) Å	Cell parameters from 2789 reflections
*b* = 15.366 (3) Å	θ = 2.5–27.9°
*c* = 7.5708 (15) Å	µ = 0.08 mm^−^^1^
β = 92.74 (3)°	*T* = 113 K
*V* = 956.4 (3) Å^3^	Block, colourless
*Z* = 4	0.32 × 0.18 × 0.12 mm

### Data collection

**Table:**

Rigaku Saturn CCD diffractometer	1681 independent reflections
Radiation source: rotating anode	1259 reflections with *I* > 2σ(*I*)
confocal	*R*_int_ = 0.040
Detector resolution: 7.31 pixels mm^-1^	θ_max_ = 25.0°, θ_min_ = 2.5°
ω and φ scans	*h* = −9→7
Absorption correction: multi-scan (*CrystalClear*; Rigaku/MSC, 2005)	*k* = −18→15
*T*_min_ = 0.974, *T*_max_ = 0.990	*l* = −8→8
6163 measured reflections	

### Refinement

**Table:**

Refinement on *F*^2^	Secondary atom site location: difference Fourier map
Least-squares matrix: full	Hydrogen site location: inferred from neighbouring sites
*R*[*F*^2^ > 2σ(*F*^2^)] = 0.035	H-atom parameters constrained
*wR*(*F*^2^) = 0.104	*w* = 1/[σ^2^(*F*_o_^2^) + (0.0648*P*)^2^] where *P* = (*F*_o_^2^ + 2*F*_c_^2^)/3
*S* = 1.03	(Δ/σ)_max_ = 0.001
1681 reflections	Δρ_max_ = 0.20 e Å^−^^3^
123 parameters	Δρ_min_ = −0.17 e Å^−^^3^
0 restraints	Extinction correction: *SHELXL*, Fc^*^=kFc[1+0.001xFc^2^λ^3^/sin(2θ)]^-1/4^
Primary atom site location: structure-invariant direct methods	Extinction coefficient: 0.109 (9)

### Special details

**Table:**

Geometry. All e.s.d.'s (except the e.s.d. in the dihedral angle between two l.s. planes) are estimated using the full covariance matrix. The cell e.s.d.'s are taken into account individually in the estimation of e.s.d.'s in distances, angles and torsion angles; correlations between e.s.d.'s in cell parameters are only used when they are defined by crystal symmetry. An approximate (isotropic) treatment of cell e.s.d.'s is used for estimating e.s.d.'s involving l.s. planes.
Refinement. Refinement of *F*^2^ against ALL reflections. The weighted *R*-factor *wR* and goodness of fit *S* are based on *F*^2^, conventional *R*-factors *R* are based on *F*, with *F* set to zero for negative *F*^2^. The threshold expression of *F*^2^ > σ(*F*^2^) is used only for calculating *R*-factors(gt) *etc*. and is not relevant to the choice of reflections for refinement. *R*-factors based on *F*^2^ are statistically about twice as large as those based on *F*, and *R*- factors based on ALL data will be even larger.

### Fractional atomic coordinates and isotropic or equivalent isotropic displacement parameters (Å^2^)

**Table:**

	*x*	*y*	*z*	*U*_iso_*/*U*_eq_	
O1	0.36719 (12)	0.93504 (6)	0.08273 (12)	0.0294 (3)	
O2	0.48038 (12)	1.05038 (6)	0.21730 (12)	0.0262 (3)	
H2	0.5244	1.0548	0.1228	0.039*	
C1	0.38084 (16)	0.98320 (8)	0.21106 (17)	0.0190 (3)	
C2	0.29007 (17)	0.97161 (8)	0.37622 (17)	0.0232 (4)	
H2A	0.2568	1.0284	0.4173	0.028*	
H2B	0.3632	0.9467	0.4669	0.028*	
C3	0.14180 (16)	0.91442 (8)	0.35423 (16)	0.0189 (3)	
C4	0.14441 (17)	0.82764 (8)	0.41446 (17)	0.0205 (3)	
C5	0.00572 (17)	0.77723 (8)	0.38957 (18)	0.0229 (4)	
H5	0.0076	0.7201	0.4303	0.027*	
C6	−0.13609 (17)	0.80882 (8)	0.30596 (17)	0.0231 (4)	
C7	−0.13687 (17)	0.89545 (8)	0.25041 (17)	0.0235 (4)	
H7	−0.2313	0.9183	0.1963	0.028*	
C8	−0.00110 (17)	0.94838 (8)	0.27356 (16)	0.0198 (3)	
C9	−0.00882 (18)	1.04182 (8)	0.21213 (18)	0.0270 (4)	
H9A	−0.1139	1.0533	0.1566	0.040*	
H9B	0.0736	1.0518	0.1289	0.040*	
H9C	0.0090	1.0798	0.3119	0.040*	
C10	0.29354 (18)	0.78874 (10)	0.50715 (19)	0.0316 (4)	
H10A	0.2726	0.7291	0.5360	0.047*	
H10B	0.3198	0.8207	0.6136	0.047*	
H10C	0.3832	0.7916	0.4308	0.047*	
C11	−0.28185 (19)	0.75097 (9)	0.2738 (2)	0.0340 (4)	
H11A	−0.2606	0.7100	0.1821	0.051*	
H11B	−0.3746	0.7858	0.2382	0.051*	
H11C	−0.3035	0.7202	0.3805	0.051*	

### Atomic displacement parameters (Å^2^)

**Table:**

	*U*^11^	*U*^22^	*U*^33^	*U*^12^	*U*^13^	*U*^23^
O1	0.0363 (7)	0.0287 (6)	0.0241 (6)	−0.0128 (5)	0.0107 (5)	−0.0080 (4)
O2	0.0285 (6)	0.0279 (6)	0.0229 (6)	−0.0113 (4)	0.0080 (4)	−0.0047 (4)
C1	0.0194 (8)	0.0168 (6)	0.0207 (7)	0.0004 (6)	−0.0010 (6)	0.0015 (5)
C2	0.0284 (9)	0.0236 (7)	0.0178 (8)	−0.0039 (6)	0.0027 (6)	−0.0005 (5)
C3	0.0243 (8)	0.0199 (7)	0.0130 (7)	−0.0031 (6)	0.0064 (6)	−0.0029 (5)
C4	0.0250 (8)	0.0212 (7)	0.0159 (7)	0.0019 (6)	0.0061 (6)	−0.0016 (5)
C5	0.0330 (9)	0.0159 (6)	0.0206 (7)	−0.0011 (6)	0.0102 (6)	−0.0005 (5)
C6	0.0259 (9)	0.0253 (7)	0.0188 (7)	−0.0049 (6)	0.0094 (6)	−0.0048 (5)
C7	0.0228 (8)	0.0295 (8)	0.0185 (8)	0.0030 (6)	0.0044 (6)	−0.0018 (5)
C8	0.0270 (8)	0.0190 (7)	0.0141 (7)	0.0001 (6)	0.0073 (6)	−0.0014 (5)
C9	0.0343 (9)	0.0233 (7)	0.0237 (8)	0.0029 (6)	0.0055 (7)	0.0014 (5)
C10	0.0317 (9)	0.0294 (8)	0.0336 (9)	0.0033 (6)	0.0022 (7)	0.0032 (6)
C11	0.0329 (10)	0.0366 (9)	0.0332 (9)	−0.0109 (7)	0.0080 (7)	−0.0040 (6)

### Geometric parameters (Å, °)

**Table:**

O1—C1	1.2222 (15)	C6—C11	1.5037 (18)
O2—C1	1.3174 (15)	C7—C8	1.3865 (18)
O2—H2	0.8200	C7—H7	0.9300
C1—C2	1.4976 (19)	C8—C9	1.5096 (18)
C2—C3	1.5067 (17)	C9—H9A	0.9600
C2—H2A	0.9700	C9—H9B	0.9600
C2—H2B	0.9700	C9—H9C	0.9600
C3—C8	1.4000 (18)	C10—H10A	0.9600
C3—C4	1.4089 (18)	C10—H10B	0.9600
C4—C5	1.3850 (18)	C10—H10C	0.9600
C4—C10	1.5080 (19)	C11—H11A	0.9600
C5—C6	1.3885 (18)	C11—H11B	0.9600
C5—H5	0.9300	C11—H11C	0.9600
C6—C7	1.3959 (19)		
			
C1—O2—H2	109.5	C8—C7—H7	119.0
O1—C1—O2	122.43 (13)	C6—C7—H7	119.0
O1—C1—C2	124.14 (12)	C7—C8—C3	119.45 (11)
O2—C1—C2	113.42 (10)	C7—C8—C9	119.81 (12)
C1—C2—C3	114.26 (10)	C3—C8—C9	120.73 (12)
C1—C2—H2A	108.7	C8—C9—H9A	109.5
C3—C2—H2A	108.7	C8—C9—H9B	109.5
C1—C2—H2B	108.7	H9A—C9—H9B	109.5
C3—C2—H2B	108.7	C8—C9—H9C	109.5
H2A—C2—H2B	107.6	H9A—C9—H9C	109.5
C8—C3—C4	119.57 (12)	H9B—C9—H9C	109.5
C8—C3—C2	119.34 (11)	C4—C10—H10A	109.5
C4—C3—C2	121.09 (12)	C4—C10—H10B	109.5
C5—C4—C3	119.05 (12)	H10A—C10—H10B	109.5
C5—C4—C10	119.27 (12)	C4—C10—H10C	109.5
C3—C4—C10	121.68 (13)	H10A—C10—H10C	109.5
C4—C5—C6	122.41 (11)	H10B—C10—H10C	109.5
C4—C5—H5	118.8	C6—C11—H11A	109.5
C6—C5—H5	118.8	C6—C11—H11B	109.5
C5—C6—C7	117.54 (12)	H11A—C11—H11B	109.5
C5—C6—C11	120.94 (12)	C6—C11—H11C	109.5
C7—C6—C11	121.52 (13)	H11A—C11—H11C	109.5
C8—C7—C6	121.95 (13)	H11B—C11—H11C	109.5
			
O1—C1—C2—C3	−18.70 (18)	C4—C5—C6—C7	−1.7 (2)
O2—C1—C2—C3	162.26 (11)	C4—C5—C6—C11	177.16 (12)
C1—C2—C3—C8	−77.82 (15)	C5—C6—C7—C8	1.3 (2)
C1—C2—C3—C4	102.58 (14)	C11—C6—C7—C8	−177.55 (12)
C8—C3—C4—C5	1.12 (19)	C6—C7—C8—C3	0.3 (2)
C2—C3—C4—C5	−179.28 (12)	C6—C7—C8—C9	−179.59 (12)
C8—C3—C4—C10	−178.12 (12)	C4—C3—C8—C7	−1.50 (19)
C2—C3—C4—C10	1.48 (19)	C2—C3—C8—C7	178.89 (11)
C3—C4—C5—C6	0.5 (2)	C4—C3—C8—C9	178.36 (12)
C10—C4—C5—C6	179.77 (13)	C2—C3—C8—C9	−1.24 (19)

### Hydrogen-bond geometry (Å, °)

**Table:**

*D*—H···*A*	*D*—H	H···*A*	*D*···*A*	*D*—H···*A*
O2—H2···O1^i^	0.82	1.84	2.6564 (15)	177

Symmetry codes: (i) −*x*+1, −*y*+2, −*z*.
